# Widespread asymmetries of amygdala nuclei predict auditory verbal hallucinations in schizophrenia

**DOI:** 10.1186/s12888-024-06301-1

**Published:** 2024-11-19

**Authors:** Magda L. Dumitru, Erik Johnsen, Rune A. Kroken, Else-Marie Løberg, Lin Lilleskare, Lars Ersland, Kenneth Hugdahl

**Affiliations:** 1 Department of Biological Sciences, University of Bergen, Thormøhlens Gate 53 A/B, Postboks 5006, Bergen, Norway; 2 Department of Biological and Medical Psychology, University of Bergen, Bergen, Norway; 3 Division of Psychiatry, Haukeland University Hospital, Bergen, Norway; 4 NORMENT Centre of Excellence, Haukeland University Hospital, Bergen, Norway; 5 Department of Clinical Engineering, Haukeland University Hospital, Bergen, Norway; 6 Institute of Clinical Psychology, University of Bergen, Bergen, Norway; 7 Department of Radiology, Haukeland University Hospital, Bergen, Norway

**Keywords:** Amygdala, Auditory verbal hallucinations, Schizophrenia, Distance index, Laterality index, Beliefs about Voices Questionnaire

## Abstract

**Background:**

Auditory verbal hallucinations, which frequently involve negative emotions, are reliable symptoms of schizophrenia. Brain asymmetries have also been linked to the condition, but the relevance of asymmetries within the amygdala, which coordinates all emotional signals, to the content of and response to auditory verbal hallucinations has not been explored.

**Methods:**

We evaluated the performance of two asymmetry biomarkers that were recently introduced in literature: the distance index, which captures global asymmetries, and a revised version of the laterality index, which captures left–right local asymmetries. We deployed random forest regression models over values computed with the distance index and with the laterality index over amygdala nuclei volumes (lateral, basal, accessory-basal, anterior amygdaloid area, central, medial, cortical, cortico-amygdaloid area, and paralaminar) for 71 patients and 71 age-matched controls.

**Results:**

Both biomarkers made successful predictions for the 35 items of the revised version of the Belief About Voices Questionnaire, such that hallucination severity increased with increasing local asymmetries and with decreasing global asymmetries of the amygdala.

**Conclusions:**

Our findings highlight a global reorganization of the amygdala, where left and right nuclei volumes differ pairwise but become proportionally more similar as hallucinations increase in severity. Identifying asymmetries in particular brain structures relevant to specific symptoms could help monitor the evolution and outcome of psychopathological conditions.

## Background

Hearing voices, also known as ‘auditory verbal hallucinations’ (AVH), is a key symptom in psychopathological conditions, including posttraumatic stress disorder, borderline personality disorder, bipolar disorder, major depression, and schizophrenia [1–4]. AVH are also common in members of the general population [5], but they are less frequent (e.g., up to 0.7% of a large sample in Britain – cf. [6]) and rarely distressful. In contrast, patients with schizophrenia report mostly negative emotional content associated with the imaginary voices they hear [7], which is likely to increase the risk for suicide [8] and, more generally, the need for care. Moreover, it is unclear why AVH are perceived as angry, menacing, harsh, malicious, derogatory, abusive, or threatening (for a review, see [9]), hence it is essential to identify clinical measures and imaging biomarkers that are reliably linked to AVH symptoms.

The goal of the current study was to investigate the potential of predicting the severity of AVH for two types of brain asymmetries, as measured by two indices: a revised version of the laterality index (‘LI’, for a review, see [10]—N.B., henceforth we use LI to refer to this version), which estimates local asymmetry, and the distance index (‘DI’, cf. [11]), which estimates global asymmetry. Specifically, we aimed to determine whether global asymmetry or mere local asymmetry of the left and the right amygdala, which is key to emotion processing, would predict the content of and response to AVH in individuals diagnosed with schizophrenia. First, we briefly review the evidence for a link between AVH and brain asymmetry in patients with schizophrenia, then we focus on amygdala asymmetry in the same clinical group, before discussing the relevance of our amygdala structure analysis in terms of local and global asymmetry to current accounts of emotional processing in patients who experience AVH.

Brain asymmetries are well documented in patients with schizophrenia, and especially in individuals suffering from AVH. The severity of the symptoms was found to be associated with abnormalities of inter-hemispheric communication [12–14] and underlying brain structures, in particular reduced hemispheric lateralization [15–17], as documented in anatomical post-mortem investigations [18–20] and MRI studies [21, 22]. Furthermore, patients with schizophrenia exhibit a shorter Sylvian fissure on the left side of the brain compared to controls (e.g., [17, 23, 24]) underscoring the importance of language-related dysfunctions in the condition [16, 25, 26].

In the amygdala, left and the right subnuclei were often found to differ in size between patients with schizophrenia and controls. Centrally located within the brain, which affords effective connections with various structures and networks [27], the amygdala coordinates all emotional signals. Importantly, although amygdala subnuclei (henceforth ‘nuclei’) are involved in processing positive content such as reward and happiness, they mostly concern negative content such as fear and anxiety (e.g., [28]). Threatening words, sounds, and images such as fearful facial expressions [29] or aversive stimuli following Pavlovian conditioning [30] increase activation in relevant sensory cortical areas, which relay information to the lateral nucleus of the amygdala [31]. Moreover, the amygdala was found to boost cortical decoding of emotional voices in sensory cortices. Damage to the amygdala impaired voice processing by the hemisphere corresponding to the side of the damage [32, 33]. Also, increased blood flow to the amygdala correlated and even generated negative emotional content in patients experiencing frequent AVH [34]. Reduced amygdala volumes were reported in patients with schizophrenia relative to controls, in particular for the evolutionarily newer basolateral group of nuclei [35] associated with fear response but also, to a lesser extent, for the evolutionarily primitive centro-corticomedial group [36, 37]. Also, Niu and colleagues [38] reported increased left-smaller-than-right amygdala volumes in male, but not in female patients with schizophrenia.

Importantly, structural changes of the amygdala, which imply abnormalities in regulating the emotional brain, may lie at the core of schizophrenia [39, 40], despite the condition being classified among ‘non-affective’ psychotic disorders according to DSM-V [41] and ICD-11 [42]. Functional MRI studies have found that the amygdala is not coding for either the intensity (arousal) or the valence of perceptual stimuli, but rather for their overall emotional value [43]. Furthermore, atypical assignment of salience to otherwise insignificant stimuli has been reported in patients with schizophrenia [44, 45], which may involve changes in neural activity not only for the amygdala, but also for other structures involved in affect regulation including the insula, the anterior cingulate cortex, and the orbitofrontal cortex, which may display reduced connectivity with the amygdala [39].

In brief, a number of studies have documented smaller amygdala volumes in patients with schizophrenia compared to controls, as well as amygdala asymmetries in patients suffering from AVH. However, these studies have evaluated exclusively the role of local asymmetries, in particular signed differences between individual nuclei in the left and the right amygdala that may be responsible for AVH. In contrast, the current study evaluates both local and global amygdala asymmetries to highlight the extent to which the severity of AVH corresponds to increased reorganization of amygdala structure in patients with schizophrenia suffering from AVH. The revised laterality index, expressed as absolute values of differences between left and right values for each amygdala nucleus, provides information on local asymmetries, whereas the distance index, expressed as absolute values of weightings between each nucleus and all other nuclei on each side, followed by a correlation between values across sides, provides information on global asymmetries. In other words, LI mainly captures differences in left–right nuclei volumes, whereas DI captures differences in overall shape, all things being equal, between the left and the right side of the amygdala. We may hypothesize that a global restructuring of the amygdala under the pressure of factors responsible for the overall shape and proportion of bodily organs [11] is driving changes in individual nuclei volumes (e.g., shrinkage or expansion of nuclei, be it to the left side, to the right side, or to both sides). If so, we should be able to observe growing changes in global asymmetry of the amygdala corresponding to ever more severe AVH symptoms, which would require simultaneous changes in local asymmetries.

## Methods

### Participants

A total of 142 adults volunteered to participate in the study. Half of them (mean age 27.58, *SD* = 9.36, 47 males) were diagnosed with auditory verbal hallucinations and schizophrenia on the ICD-10 [46]. The other half were healthy controls, which self-reported the absence of any (neuro)psychological conditions. Controls were individually matched for age within 3 years distance on average. Diagnoses were based on the DSM-IV Axis I Disorders (SCID-I) structured clinical interview [47], which was conducted by trained physicians/psychiatrists and psychologists, and were subsequently converted to ICD-10 diagnoses. A further inclusion criterion for patients was a score of 3 or higher on the third item of the Positive and Negative Syndromes Scale (PANSS) [48], which is widely used to assess the presence of AVH. Each volunteer provided written informed consent before participating in the study, according to the Regional Committee for Medical Research Ethics in Western Norway (REK Vest #2016/800), which approved the study, and the Declaration of Helsinki.

### Clinical assessment

Hallucination severity was assessed with the P3 item of the PANSS, which was further evaluated by the physicians/psychiatrists performing the interview. Patients diagnosed with any of the subcategories of schizophrenia were included in the study, as long as they had at least ‘mild’ AVH (P3 score of 3 or higher), to better assess the relevance of amygdala structural changes. Severity in PANSS is assigned levels from 1 to 7, which correspond, respectively, to ‘absent’, ‘minimal’, ‘mild’, ‘moderate’, ‘moderate severe’, ‘severe’, and ‘extreme’ hallucinatory behavior. Medication used at the time of the scan was converted into defined daily dose (DDD) for each patient and averaged across groups. A total of 53 patients were using second generation antipsychotics (DDD = 0.804), of which 6 also used first-generation antipsychotic medication (DDD = 1.085). There were also patients who used, anti-depressants (*n* = 10), benzodiazepines (*n* = 18), anticholinergics (*n* = 5), and mood stabilizers (*n* = 2) in addition to second-generation antipsychotics. Data from 2 patients was insufficient, accounting for slight deviations in overall counts. Mean illness duration for the patients was 4.3 years (*SD* = 7.7). Mean positive PANSS scores were 17.98 (*SD* = 4.44) with a mean P3 score of 4.43 (*SD* = 0.86), mean negative PANSS scores were 16.95 (*SD* = 5.77), and mean general psychopathology PANSS scores were 34.53 (*SD* = 9.08).

Perceived AVH content was assessed using the revised version of the Beliefs about Voices Questionnaire [49]. PANSS and BAVQ-R scores were collected on the same day and one week before the MRI scan respectively. The BAVQ [50] is a clinical instrument for self-evaluation, on a four-point scale from ‘disagree’ (0) to ‘strongly agree’ (3), of key aspects of beliefs about voices along three subscales: Malevolence (i.e., the perceived negative intent that individuals attribute to imaginary voices), Omnipotence (i.e., the power that voices are perceived to have over patients’ lives), and Benevolence (i.e., the perceived positive intent of imaginary voices). In brief, beliefs about the content of imaginary voices highlight the patients’ perception of, rather than the actual content of voices. The BAVQ-R is a revised version of the BAVQ, which includes four supplementary subscales to measure Engagement versus Resistance responses to voices, each of them further separated into emotional and behavioral modes of expression. A study examining the factor structure of the BAVQ-R supports a simplified, two-factor model opposing benevolence to Malevolence and Omnipotence combined [51]. Moreover, the authors found no evidence for separating behavioral from emotional response types, but underscore the importance of distinguishing between Resistance to voices versus Engagement. Indeed, therapeutic approaches are more successful if they reduce the urge to resist voices, which was found to increase anxiety and depression [49].

### MRI data acquisition and preprocessing

MR data were acquired using a 3 T GE 750 scanner at the Haukeland University Hospital in Bergen, Norway, contingent to clinical data acquisition. A structural T1-weighted image was acquired (7.42 min) using a 3D SPGR sequence with the following parameters: TR/TE/FA/FOV 7.78 ms/2.94 ms/14°/ 256 mm (post-upgrade: 6.9 ms/3.0 ms/14°/256 mm), isotropic voxel size of 1mm3. T1-weighted MRI volumes were preprocessed with the *Freesurfer* image analysis suite (v 6.0.0, http://surfer.nmr.mgh.harvard.edu/) [52]. Automated segmentation of the bilateral amygdala and all amygdala nuclei was performed using a probabilistic atlas [53] based on ultra-high-resolution ex vivo MRI data (~ 0.1 mm isotropic).

### Raw volumes

In a series of *t*-tests, we compared the raw volumes of nine nuclei to the left and the right side of nine amygdala: lateral, basal, accessory-basal, anterior amygdaloid area, central, medial, cortical, cortico-amygdaloid area, and paralaminar.

### Computing the distance index (DI) and the revised laterality index (LI)

Next, we computed the distance index and the laterality index by combining the left and the right side for each nucleus, as detailed in Dumitru [11] and entered each set of values as predictors of BAVQ-R scores for each set of 35 items in random forest regression models. The factor ‘group’ (i.e., patients versus controls) was added to each model. We obtained nine DI values for the nine left and right amygdala nuclei by first calculating absolute differences between the volume of each nucleus and the volumes of the remaining eight nuclei on each side, resulting in two measurement vectors, one for the left, the other for the right side. Pearson correlation coefficients were subsequently derived for each nucleus and subtracted from 1. The higher the DI value, the greater should be the difference between the left and the right side of a nucleus. Next, we computed LI values, one for each amygdala nucleus, and derived absolute differences. The higher the LI values, the greater should be the difference in size between the sides of a nucleus.

### Random forest regression models

To evaluate the performance of LI and DI models, we correlated model-predicted scores with actual scores on each of the 35 BAVQ-R items. Importantly, models were run over all amygdala nuclei simultaneously, which impacts the interpretation of our results, as follows. Good performance by local LI models and bad performance by global DI models would suggest that differences between patients and controls must be traced back to differences in amygdala nuclei size between the left and the right side that are proportional, such that the overall shape of the left and the right amygdala remains unchanged in patients versus controls. In contrast, poor performance by LI models and good performance by DI models would suggest that differences between patients and controls concern the overall shape of the two amygdala sides. Higher DI values in patients compared to controls could occur for the whole amygdala when the volume of a single nucleus differs between groups. In contrast, pairwise differences for several amygdala nuclei need to occur for overall high LI values. Another possible outcome when comparing DI and LI models is good AVH prediction across the board. In this case, asymmetry is widespread, as there should be several individual nuclei that differ in volume between the left and the right side and the relationship between them that is, the overall amygdala shape for the left and the right side, should differ across patients and controls.

Statistical analyses were performed in the statistical environment R, version 2022.07.2 [54]. We split the data into a training set (80%) and a test set (20%) and ran random forest regression models for each set of index values (DI and LI) and for each BAVQ item. Models included nine index values corresponding to the nine nuclei. By default, controls were assigned zero values to all items. The factor ‘group’ (patients versus controls) was included in each model. Models were fitted using the best estimators, and the optimized models were applied to the test sample. We further applied tenfold grid leave-one-out cross-validation over test data and report R^2^ and RMSE to evaluate prediction accuracy in the test set. Predicted and actual BAVQ-R scores were further correlated for each of the 35 DI models and the 35 LI models along the seven subscales ‘Malevolence’, ‘Omnipotence’, ‘Benevolence’, ‘Behavioral resistance’, ‘Emotional resistance’, ‘Behavioral engagement’, and ‘Emotional engagement’.

### Partial dependence plots

We built dependence plots to visualize the relationships the model has learned between response and predictors. These plots summarize the marginal effect one predictor that is, the volumes of individual DI or LI nucleui, has on the outcome of a regression model.

## Results

### Raw volume asymmetries

**Fig. 1:**
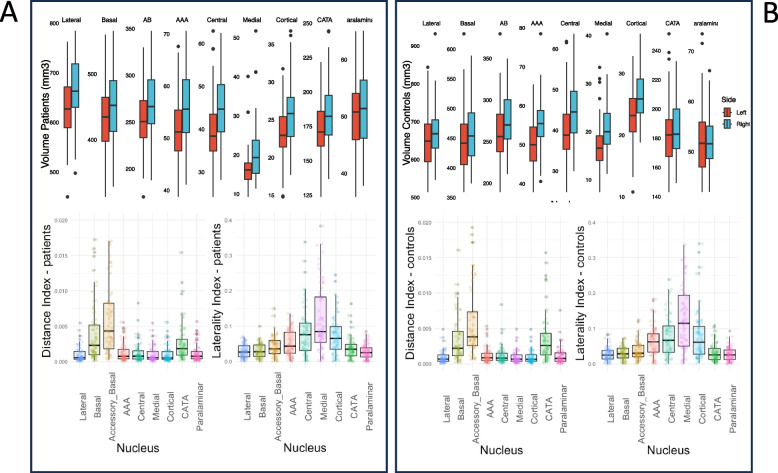
Raw values and index values of amygdala nuclei in patients (**A**) and controls (**B**). Right nuclei are often significantly greater than left nuclei. The distance index is greatest for large nuclei including the accessory basal, basal, and CATA. The laterality index is largest for the smallest nuclei including the medial, central, and cortical

Overall, raw left–right asymmetries of amygdala nuclei were larger for patients compared to controls. We examined raw volume values by computing paired *t*-*tests* and *Cohen’s d* measures for each pair of left and right anatomical amygdala nuclei to identify raw asymmetries. In patients, the left side was significantly smaller than the right side for all but one amygdala nucleus, as seen in Fig. 1(A): lateral (*p* = 0.004, *d* = 0.49), basal (*p* = 0.03, *d* = 0.37), accessory-basal (*p* < 0.001, *d* = 0.60), AAA (*p* < 0.001, *d* = 0.65), central (*p* < 0.001, *d* = 0.70), medial (*p* < 0.001, *d* = 0.71), cortical (*p* < 0.001, *d* = 0.76), and CATA (*p* = 0.001, *d* = 0.53), paralaminar (*p* = 0.274, *d* = 0.18). In controls, the left side of the amygdala was smaller than the right side in six out of the nine nuclei, as seen in Fig. 1(B), as follows: lateral (*p* = 0.039, *d* = 0.35), basal (*p* = 0.102, *d* = 0.27), accessory-basal (*p* = 0.009, *d* = 0.44), AAA (*p* < 0.001, *d* = 0.80), central (*p* < 0.001, *d* = 0.67), medial AAA (*p* < 0.001, *d* = 0.67), cortical (*p* < 0.001, *d* = 0.75), CATA (*p* = 0.29, *d* = 0.18), Paralaminar (*p* = 0.787, *d* = 0.04). In brief, left–right raw asymmetry appears to be stronger in patients compared to controls.

### Index values

Based on the raw volumes of amygdala nuclei, we computed global and local index values, DI and LI respectively, which we subsequently entered as predictors of the 35 BAVQ scores in random forest models. Figure 1 summarizes DI and LI values computed for each pair of amygdala nuclei. The largest asymmetry values in both patients and controls were obtained for the DI-computed accessory-basal, basal, and CATA nuclei, and for the LI-computed medial, central, and cortical nuclei for LI. Incidentally, average raw values for amygdala nuclei, both left and right, are among the highest for the DI-computed nuclei, and the lowest for the LI-computed nuclei mentioned.

### BAVQ-R scores

The patients with schizophrenia in our study experienced mostly negative AVHs, as they scored negative BAVQ-R items higher than positive items. Table 1 summarizes scores (mean and standard deviation) for all 35 BAVQ-R items and for the 71 patients organized over seven subscales (shaded rows correspond to positive values). A brief examination shows that negative subscales scored up to three times higher than positive subscales.

**Table 1:** Patient responses to BAVQ-R items (mean and SD). Shaded areas cover positive-valence items

Subscale	BAVQ item	Mean	SD
Malevolence	1. My voice is punishing me for something I have done	1.348	1.223
	4. My voice is persecuting me for no good reason	1.614	1.219
	7. My voice is evil	1.449	1.195
	10. My voice wants to harm me	1.366	1.198
	13. My voice wants me to do bad things	1.183	1.223
	16. My voice is trying to corrupt or destroy me	1.380	1.269
Omnipotence	3. My voice is very powerful	1.507	1.217
	6. My voice seems to know everything about me	1.986	1.222
	9. My voice makes me do things I really don't want to do	1.268	1.183
	12. I cannot control my voices	2.113	1.063
	15. My voice will harm or kill me if I disobey or resist it	0.783	1.055
	18. My voice rules my life	1.206	1.087
Behavioral resistance	27. I tell it to leave me alone	1.714	1.218
	28. I try and take my mind off it	2.113	1.036
	29. I try and stop it	1.746	1.105
	30. I do things to prevent it talking	1.771	1.079
	31. I am reluctant to obey it	1.710	1.139
Emotional resistance	20. My voice frightens me	1.571	1.162
	22. My voice makes me feel down	1.915	1.038
	23. My voice makes me feel angry	1.549	1.144
	25. My voice makes me feel anxious	1.643	1.130
Benevolence	2. My voice wants to help me	1.043	1.042
	5. My voice wants to protect me	1.014	1.042
	8. My voice is helping to keep me sane	0.775	1.058
	11. My voice is helping me to develop my special powers or abilities	0.577	0.951
	14. My voice is helping e to achieve my goal in life	0.725	0.998
	17. I am grateful for my voice	0.757	0.999
Behavioral engagement	32. I listen to it because I want to	0.800	0.894
	33. I willingly follow what my voice tells me to do	0.592	0.888
	34. I have done things to start to get in contact with my voice	0.577	0.936
	35. I seek the advice of my voice	0.657	0.961
Emotional engagement	19. My voice reassures me	0.725	0.983
	21. My voice makes me happy	0.479	0.843
	24. My voice makes me feel calm	0.580	0.898
	26. My voice makes me feel confident	0.443	0.792

### Random forest regression models

**Fig. 2:**
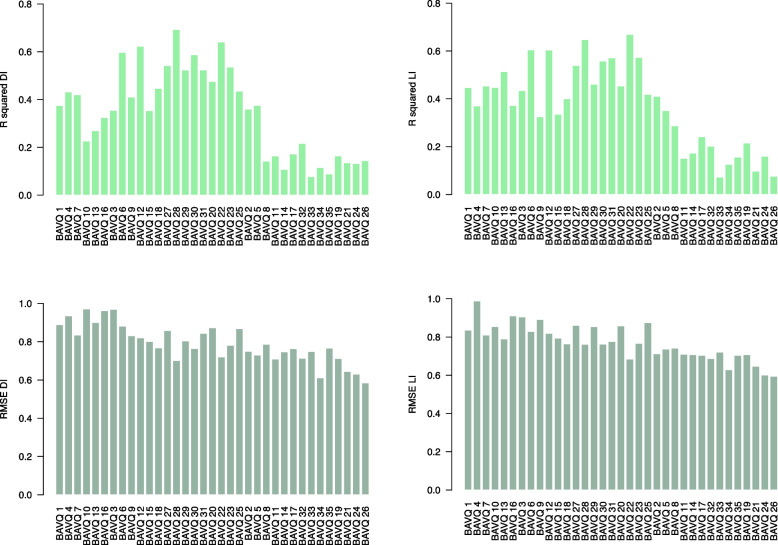
Average *R*^*2*^ and root mean square error (*RMSE*) for each of the 35 BAVQ-R prediction models with DI- and LI-computed amygdala nuclei as predictors. BAVQ-R items are scored on a four-point scale from ‘disagree’ (0) to ‘strongly agree’ (3) and are grouped into four negative subscales (items 1 to 25) followed by three positive subscales (items 2 to 26). *R*^*2*^ values are much smaller for positive than for negative subscales

The models allowed us to determine that both local and global asymmetries between left and right amygdala nuclei are relevant to the frequency and/or intensity of AVH measured with the BAVQ-R. For each set of the 35 random forest regression models (one set for LI and another for DI), corresponding to the 35 BAVQ-R items, we obtained *RMSE* and* R*^*2*^ values, as shown in Fig. 2. A brief examination of the values across BAVQ-R subscales reveals good *RMSE* performance but a general tendency towards lower *R*^*2*^ scores for positive subscales (Benevolence, Behavioral engagement, and Emotional engagement) than for negative subscales (Malevolence, Omnipotence, Behavioral resistance, and Emotional resistance).

**Fig. 3:**
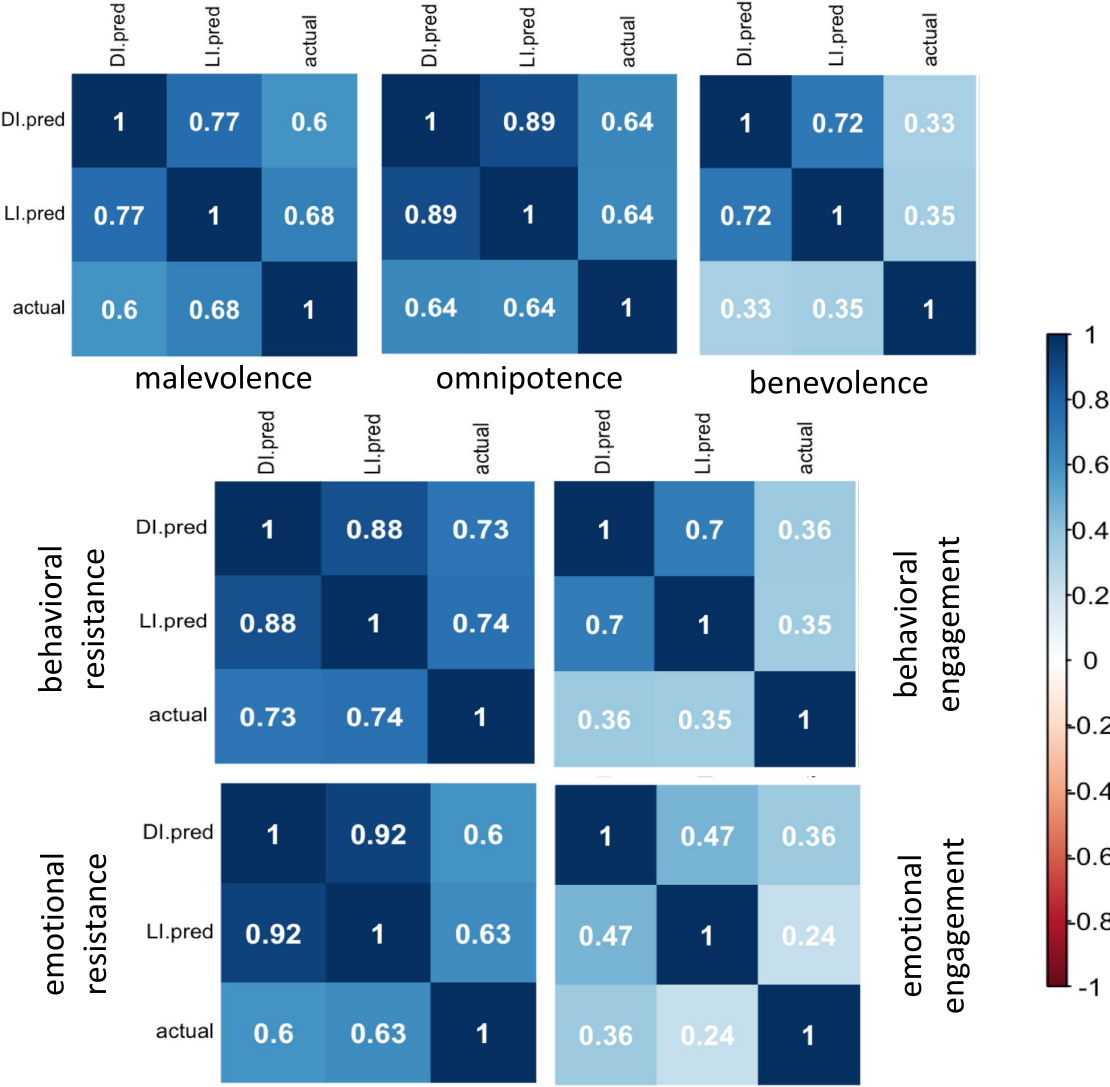
Correlation matrices for predicted versus actual responses to BAVQ-R items. Correlation coefficients are higher for negative subscales (Malevolence, Omnipotence, Behavioral resistance, and Emotional resistance) compared to positive subscales (Benevolence, Behavioral engagement, and Emotional engagement) across DI- and LI- models

To compare model-predicted and actual responses to BAVQ items, we compiled all observations into the seven BAVQ-R subscales before correlating them. Figure 3 summarizes the correlation coefficients, which were all positive and significant below the 0.001 *p*-level for DI as well as for LI models, except for the LI emotional engagement subscale, whose *p*-value was 0.011. Overall, correlation coefficients were lower for positive subscales than for negative subscales.

### Partial dependence plots

Partial dependence plots revealed which responses to BAVQ items are most relevant to specific asymmetries between amygdala nuclei. Overall, negative subscales scores were associated mostly with DI values for accessory basal, basal, and AAA nuclei, but also with paralaminar, and CATA nuclei, and mostly with LI values for accessory-basal, basal, and CATA nuclei, but also with paralaminar, central, medial, and AAA nuclei. Positive subscales scores were associated with DI values for central, medial, and AAA nuclei, but also with CATA, accessory-basal, cortical, and paralaminar nuclei, and mostly with LI values for basal and CATA nuclei, but also with lateral, medial, central, and paralaminar nuclei.

**Fig. 4:**
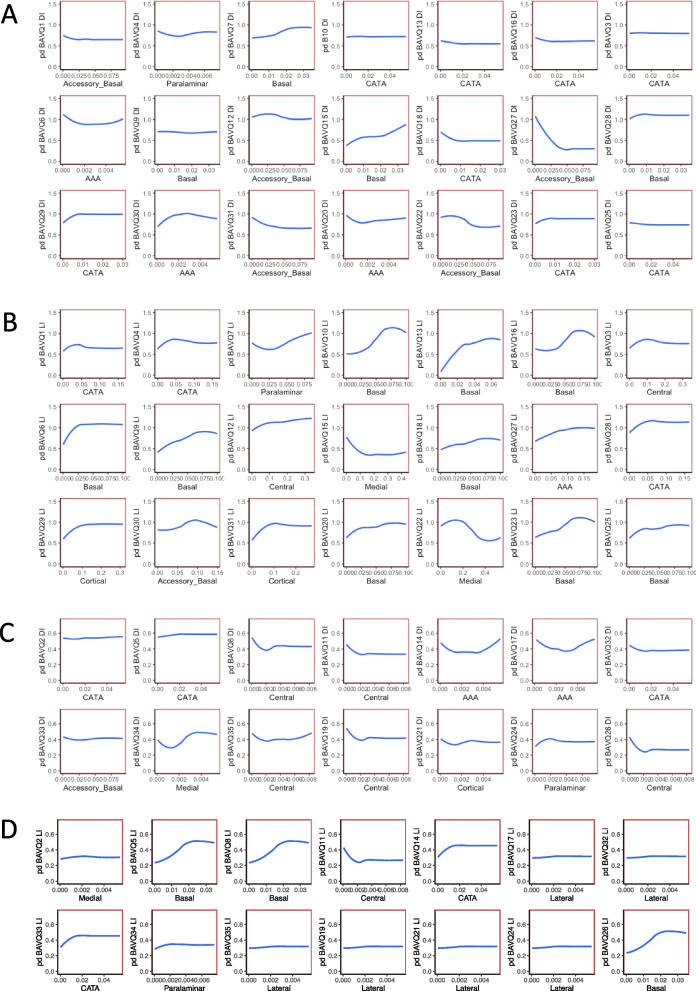
Partial dependence plots of random forest regression models. Marginal effects for each amygdala nucleus ranking highest in importance for each model were computed as predictors of BAVQ-R scores (BAVQ, in short) in DI and LI regression models. Predicted DI or LI scores are given on the y-axis, whereas predicted scores are given on the x-axis. For negative and positive subscales of DI models (**A** and **C**), many plots are descending (i.e., the higher the BAVQ-R scores, the smaller the global asymmetry) whereas for negative and positive subscales of LI models (**B** and **D**), many plots are ascending (i.e., the higher the BAVQ-R scores, the higher the local asymmetry)

The hierarchy of predictors – here, local and global index values computed over the volumes of amygdala nuclei – specifies the percentage of their contribution to the model. The direction of this contribution can be visualized using partial dependence plots. We compiled a list of the highest ranked nucleus for each DI and LI model and obtained dependence plots for the regression models we fitted them to predict BAVQ-R scores. The plots visualize the relationships the model has learned between global or local asymmetries of amygdala volumes and AVH. As shown in Fig. 4, plots are grouped by negative subscales across DI models (A) and across LI models (B), and by positive subscales across DI models (C) and across LI models (D). To take one example, the first plot in section A illustrates the impact of the DI-computed accessory-basal nucleus volume on item 1 of the BAVQ-R scale, showing higher BAVQ 1 scores for low DI values, with performance plateauing rapidly at higher DI values. Indeed, most DI partial dependence plots for both negative and positive BAVQ-R subscales show a similar pattern, suggesting that AVH are strongest and especially most distressful when the two sides of the amygdala have similar shapes that is, are globally similar hence are associated with low DI values. In contrast, most LI partial dependence plots, for example item 7 under B, show that higher BAVQ-R scores are associated with higher LI values, indicating that AVH are stronger and more distressful when the two sides of the amygdala are locally asymmetric.

## Discussion

We have shown that the degree of similarity between the left and the right side of the amygdala in terms of relative proportions among nuclei could predict the severity of auditory verbal hallucinations in patients with schizophrenia. In other words, models including local and global indices as predictors, which we computed based on raw amygdala volumes, yielded predicted BAVQ-R scores that were positively and significantly correlated with actual scores. Our findings reveal that the degree of global amygdala asymmetry, which involves one or several instances of local asymmetry between individual left–right nuclei, is a successful biomarker of AVH. These findings further illuminate the amygdala role in emotion response and processing, including possible reasons for aberrant network connectivity with other brain regions relevant to emotion regulation and highlight the potential of an analysis based on local versus global structural asymmetry to model the experience of AVH and other psychopathology symptoms. We evaluate these implications of our findings below.

Both local and global models captured by LI and DI indices, as described in Dumitru [11], were successful predictors of the content of and response to AVH. The reason why we need to assess both global and local amygdala asymmetry is that the latter alone cannot be a robust predictor of AVH that is, LI cannot distinguish between proportional and idiosyncratic changes in nuclei volumes. For example, left nuclei could all be ten percent smaller in patients compared to controls, which does not alter the overall ‘shape’ (i.e., the pattern of proportional changes in nuclei volumes) of the left amygdala compared to the right that is, global amygdala asymmetry. Alternatively, left nuclei could be smaller, each to a variable extent, which could alter the shape of the left amygdala compared to the right that is, global amygdala asymmetry. In brief, global changes require local changes, whereas local changes do not require global changes. Finding robust predictions of LI and DI models amounts to observing global changes in amygdala structure in patients with schizophrenia suffering from AVH.

Our findings thus support previous evidence for brain structure alterations in patients with schizophrenia [23, 35, 55–58]. For example, hemispheric specialization has been linked to neurophysiologic, neurobehavioral, and neuroanatomical measures [59–61], such that AVH symptoms are associated with damage to the left temporal lobe and language-related regions [62]. Furthermore, by highlighting the relevance of amygdala asymmetries to AVH, these findings underscore the link between schizophrenia symptoms and the emotional brain, given the well-established involvement of the amygdala in emotion processing [28, 63], specifically in emotional language processing [33, 34]. We showed that mostly negative rather than positive content and responses to AVH are well predicted by asymmetries in amygdala volumes. In addition, raw volumes of amygdala nuclei were more asymmetric in patients (eight nuclei were smaller to the left than to the right in patients, compared to only six nuclei in controls) and *R*^*2*^ values were higher for the negative BAVQ-R subscales Malevolence, Omnipotence, Behavioral resistance and Emotional resistance than for the positive subscales Benevolence, Behavioral engagement, and Emotional engagement. An implied lesser role of changes in amygdala structure for positive compared to negative emotions is in line with the results of a BAVQ-R factor analysis showing that Benevolence must be distinguished from Malevolence and Omnipotence combined, and that Behavioral and Emotional response types should be conflated, while distinguishing resistance to voices from engagement with voices [51]. Indeed, Pearson correlations between DI or LI model predictions and actual BAVQ-R scores were clustered along two axes, as follows: correlation coefficients were higher for subscales measuring negative content and resistance to voices than for subscales measuring positive content and engagement with voices. We may assume that global amygdala changes in patients suffering from AVH that is, a tendency towards greater uniformity of proportions among nuclei volumes as symptoms increase in severity, are indicative of ample reorganization of the amygdala, with pairwise volume differences preceding global fine-tuning of differences between the left and the right amygdala.

Furthermore, together with structural changes in other brain areas relevant to emotion regulation, amygdala asymmetries in patients suffering from AVH may underlie aberrant neural patterns of activation and connectivity [39]. Indeed, structural changes in the amygdala are thought to underly deficits in emotional perception and behavior as well as aberrant emotional reactivity [39, 40], which ultimately alter the connections with the insula, the anterior cingulate cortex, and the orbitofrontal cortex. Reduced connectivity of the amygdala with these areas can be explained by a series of changes in local structures, such that the sizes of left- and the right-side nuclei become, overall, more similar to each other in terms of their proportions within each side. Indeed, an examination of dependence plots for the highest ranked nuclei in terms of contribution to DI and LI models reveals that local and global asymmetry changed in opposite directions that is, BAVQ-R scores increased with increasing local asymmetry but decreased with increasing global asymmetry.

Although changes in brain structure are more likely to trigger changes in brain function, rather than the other way around, the opposite can also be true. Brain asymmetry could have occurred as an adaptive response to stressors, ultimately providing important processing advantages [63]. The authors review evidence suggesting a link between structural brain asymmetries, functional lateralization, and HPA-axis function, with specific relevance to psychiatric and neurodevelopmental disorders. For example, left amygdala volumes were smaller in patients with post-traumatic stress disorder (PTSD) compared to healthy controls, which boosts the overall dominance of the right hemisphere in processing negative emotions, which are triggered by stressful situations. These findings support a greater involvement of the right hemisphere in perception and cognition as an adaptive response in patients with PTSD. In our study, amygdala nuclei were smaller to the left than to the right in patients with schizophrenia, leading to widespread local asymmetries. It is thus conceivable that structural amygdala changes occur as compensatory effects to distressful symptoms in schizophrenia.

Importantly, our analysis may impact the way that research is carried out when attempting to identify a link between brain structure and psychopathology symptoms – in our case, between amygdala structure and AVH symptoms in patients with schizophrenia compared to controls. Previous studies have yielded inconclusive evidence (for reviews, see [55, 56]), possibly because the usual methodology was upheld that compared raw volumes or computed, one by one, signed laterality values of brain sub-structures. In contrast, by investigating which type of structural asymmetry is relevant to AVH that is, whether local asymmetry alone or rather global asymmetry is a better predictor, we demonstrated that an analysis in terms of structure type rather than individual values can yield good predictions of AVH symptoms. Moreover, by including all amygdala nuclei in our analysis, we are likely to capture all relevant dimensions of AVH, which are generated through and sustained by a network of low-level sensory information and neuroendocrine modulation systems, as well as by higher-level cognitive modules involving fear conditioning, language, and memory. The upshot of a comprehensive analysis of structural amygdala changes linked to AVH in schizophrenia patients is to achieve a better and more timely understanding of schizophrenia symptoms based on imaging data, which may improve symptom management in high-risk individuals.

In terms of limitations of the current study, perhaps the most important is the way we defined global asymmetry as absolute values, which does not distinguish between biases to the left or to the right. We acknowledge the lack of such information as a necessary tradeoff for computing the overall magnitude of local and global asymmetries. However, there are limitations of our study that we can addressed in future research. An investigation of sex differences in terms of AVH severity linked to amygdala structure would illuminate the genetic or hormonal impact on the growth patterns responsible for structural changes. Also, it would be interesting to investigate disease duration in conjunction with the type of second-generation antipsychotics administered to patients in order to determine whether structural changes in the amygdala are epiphenomena of medication choice. Finally, an assessment could be carried out to determine whether early detection of global asymmetries can mitigate the management of high-risk cases and the need for care.

## Data Availability

The datasets analyzed during the current study are not publicly available due to a requirement by Norwegian Regional Committee for Medical Research Ethics for data to remain confidential, but are available after being de-identified from the corresponding author on reasonable request.
